# Development of Adapalene Loaded Liposome Based Gel for Acne

**DOI:** 10.3390/gels9020135

**Published:** 2023-02-06

**Authors:** Asma Arooj, Asim Ur Rehman, Muhammad Iqbal, Iffat Naz, Aiyeshah Alhodaib, Naveed Ahmed

**Affiliations:** 1 Department of Pharmacy, Quaid-i-Azam University, Islamabad 45320, Pakistan; 2 Drug Delivery and Cosmetic Lab (DDCL), Faculty of Pharmacy, Gomal University, Dera Ismail Khan 29050, Pakistan; 3 Department of Biology, Science Unit, Deanship of Educational Services, Qassim University, Buraydah 51452, Saudi Arabia; 4 Department of Physics, College of Science, Qassim University, Buraydah 51452, Saudi Arabia

**Keywords:** adapalene, optimization, particle size, animal model, liposomes, Carbopol

## Abstract

Retinoids are considered the mainstay treatment for moderate to severe acne. Adapalene, a third-generation retinoid, has physiochemical properties which hinder the effective delivery of the drug to the skin. Therefore, the current study aimed to develop and evaluate adapalene liposomal loaded gel (ADA-LP gel) for the effective management of acne to improve tolerability and delivery to targeted sites as compared to the conventional dosage form of the drug. A novel spontaneous phase transition method (SPT) was used to formulate liposomes. Liposomal formulation (ADA-LP) was prepared and optimized based on particle size, zeta potential, and PDI. Optimized formulation was further characterized by different techniques and loaded into Carbopol gel. In vitro drug release, ex vivo permeation, and in vivo studies were performed using the prepared adapalene-loaded liposomal-based gel. The in vivo study was done employing the testosterone-induced acne model in mice. The optimized formulation had a size of 181 nm, PDI 0.145, and a zeta potential of −35 mV, indicating that the formulation was stable. Encapsulation efficiency was 89.69 ± 0.5%. ADA-LPs were loaded into the gel. Prepared ADA-LP showed a 79 ± 0.02% release of drug in a sustained manner, within 24 h. The ex vivo permeability study showed a total of 43 ± 0.06 µg/cm^2^ of drug able to permeate through the skin within 24 h. Moreover, only 28.27 ± 0.04% was retained on the epidermis. The developed ADA-LP gel showed significant improvement in the acne lesions in mice with no visible scars and inflammation on the skin. Therefore, ADA-LP-based gel could be a promising carrier system for the safe and effective delivery of Adapalene.

## 1. Introduction

Acne vulgaris (AV) is an inflammatory skin condition affecting 9.38% of the world population which makes it the 8^th^ most prevalent disease globally [1]. Skin lesions are a characteristic feature and can be both inflammatory and non-inflammatory in nature. The quality of life (QoL) of patients with severe acne is comparable to the quality of life of patients with asthma, arthritis, or epilepsy [2]. In recent years, nanotechnology was used to decrease side effects, improve tolerability, and protect drugs from degradation. Polymeric and lipid-based nanoparticles have been investigated widely. However, lipid-based nanoparticles have the advantage of being biocompatible, non-toxic, and superior to polymeric nanoparticles [3]. Liposomes (LP) are the most commonly used and studied nanoformulation [4]. The spontaneous transition method (SPT) was first described in 2020. The simplicity, shorter processing time, and reproducibility make this method superior to most of the other methods used currently [5].

Treatment options recommended by the American Academy of Dermatology Association (AAD) include antibiotics such as clindamycin and erythromycin, benzoyl peroxide, and retinoids. Increased resistance to antibiotics limits their use [6]. Benzoyl peroxide is associated with allergic contact dermatitis [7] and even facial edema [8]. Retinoids are vitamin A derivatives and are a 1st line treatment for AV. Adapalene (ADA) is third generation retinoid, approved in 1996 for clinical use. It is a synthetic naphthoic acid derivative. Adapalene regulates the cell turnover and differentiation of keratinocytes in a series of steps. Adapalene also regulates inflammatory processes. It inhibits the formation of prostaglandins by reducing the activity of lipoxygenase and metabolism of arachidonic acid [9]. Adapalene has a pKa value of 4.00, and thus it can act as a skin irritant. Therefore, the conventional dosage form of ADA is associated with side effects such as redness, irritation, etc. Adapalene is also highly lipophilic with a log *p* value of 8, and the physiochemical properties of ADA reduce its effectiveness and tolerability even after being incorporated into conventional gel form. These side effects limit the topical use of ADA [10].

It was reported that the pH of the skin in the acne vulgaris increases [11,12]. Acidic pH allows micro-flora to flourish but as the pH increases, changes in the growth of this micro-flora occurs [13]. Evidence suggests that the acidic pH inhibits the growth of acne caused by bacteria, *Propionibacterium acnes*, and their growth significantly increases in the pH range of 6.0–6.5 [14]. The work plan was designed to effectively deliver ADA at the pH of acne lesions, that is, 6.4. The current study is focused on the development of adapalene-loaded liposomes (ADA-LP) incorporated into the gel. Liposomes of adapalene were reported in the literature. Kumar and Banga (2015) prepared liposomes of ADA using a thin film hydration method, and liposomes were further characterized and studied. However, the prepared liposomes were neither incorporated nor studied into a patient-friendly dosage form [15]. Here, we report the optimization, characterization, and in vivo analysis of adapalene liposomes prepared by a novel SPT method and further incorporated in Carbopol 934 gel in order to combat problems associated with the physiochemical properties of drug.

## 2. Results and Discussion

### 2.1. Optimization of LPs

The summary of the design is given in Table 1. Twenty-nine runs were formulated as generated by Design Expert^®^ (version 12.0.). Data was analyzed in terms of responses after the evaluation of the significance of the model through ANOVA, with a *p*-value < 0.05. Run 22 was picked by the software as the optimized formulation.

Different factors were varied, and responses were noted with respect to each run are given in Table 2. The particle size, zeta potential, and polydispersity index (PDI) of blank liposomes (LP) were studied to evaluate the properties.

#### Effect of Various Factors on the Responses of Particle Size, Zeta Potential, and PDI

**Figure 1:**
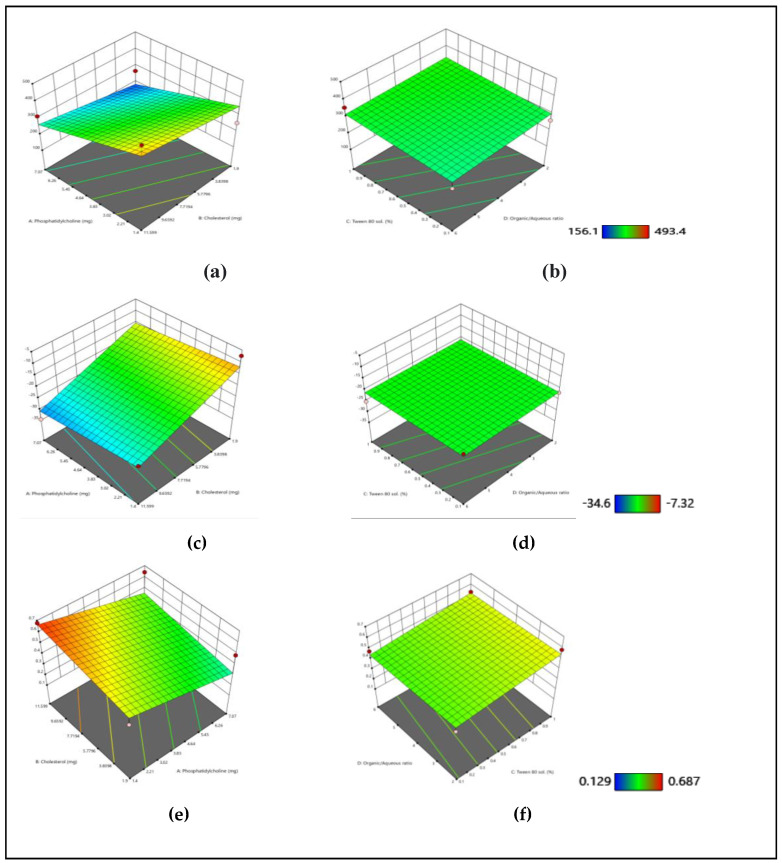
3D graphical representation of variables including PC, cholesterol, Tween 80 solution (%), and organic to aqueous ratio on responses particle size (**a**,**b**), zeta potential (**c**,**d**), and PDI (**e**,**f**).

The effect of each factor on particle size, zeta potential, and PDI are depicted in Figure 1. The model for each response was significant with a *p*-value < 0.05. The effect of different factors on each response, however, was different, and here, the results are discussed where different factors had a significant effect (*p* value < 0.05) on each response. The effect of variables on particle size is shown in Figure 1a,b. Increase in the concentration of cholesterol leads to an increase in size. Cholesterol interacts with the hydrophobic phosphatidylcholine chains via hydrogen bonding. Hydrophobicity and structure of cholesterol allows it to readily insert itself in between the lipid bilayers. Therefore, the concentration of cholesterol is directly linked to the increase in size [16]. The particle size of liposomes increased with the increase in the organic to aqueous ratio. The organic phase was kept constant, and thus the increase in the ratio resulted in an increase in the aqueous core of liposomes, which ultimately increased the particle size [5]. The effect of variables on zeta potential is displaced in Figure 1c,d.

An increase in the concentration of lipid and organic to aqueous ratio, and a decrease in the concentration of Tween 80 resulted in the formation of liposomes with more positive zeta potential. PC has a positively charged choline group, and thus increased concentration of PC resulted in the formation of liposomes with a positive charge [17]. However, an increase in the concentration of cholesterol shifted the zeta potential towards negative. It can be explained by the fact that cholesterol has a negative charge, and increased concentration leads to an even more negative charge [18].

Similarly, PDI increased as the concentration of lipid, Tween 80, and the organic to aqueous ratio increased, as shown in Figure 1e,f. PDI is a measurement of stability of the nanocarrier system. It describes the heterogeneity of nanoparticles based on the size. A lower value indicates that the system is homogenous and thus stable. At a higher concentration of surfactant (Tween 80), there is an abundance of free micelles in the system causing flocculation which results in an increase in PDI [19]. Cholesterol provides stability to the prepared vesicles. Hence, an increase in the concentration of cholesterol decreased PDI. A low solid to solvent ratio in the organic phase produces liposomes of low PDI as reported in the literature [5]. Hence, increasing the concentration of lipid increased PDI.

### 2.2. Preparation of Optimized Blank and ADA-Loaded Liposomes

The optimized blank formulation was prepared after the evaluation responses of all formulations. The optimized formulation was prepared with 7.07 mg of PC, 6.7495 mg of cholesterol, 0.1% Tween 80 solution, and a 1:4 organic to aqueous ratio. Drug-loaded liposomes were prepared using optimized factors and 1.2 mg of ADA using the modified SPT method.

#### Characterization of LPs and ADA-LP

**Figure 2:**
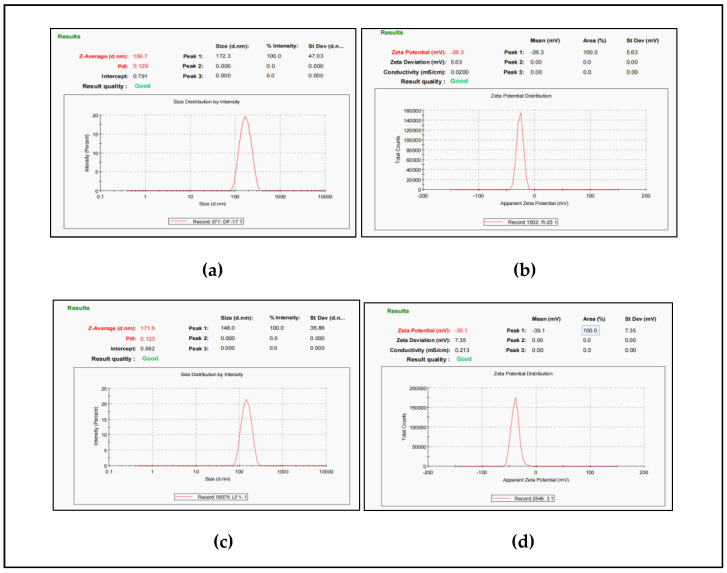
(**a**) Particle size and PDI of LPs (**b**) Zeta potential of LPs. (**c**) Particle size and PDI of ADA-LP (**d**) Zeta potential of ADA-LPs.

The particle, zeta potential, and PDI of blank liposomes were 156.7 ± 2.6 nm, −26.3 ± 1.5 mV, and 0.129 ± 1.3, respectively, as shown in Figure 2a and 2b. The particle size, zeta potential, and PDI of ADA-LPs were 171.5 ± 4.1 nm, −35 ± 2.1 mV, and 0.123 ± 1.7, respectively, as shown in Figure 2c and 2d. The significant increase in the zeta potential can be explained by the presence of negatively charged groups (–COOH) present in the ADA [20]. 

**Figure 3:**
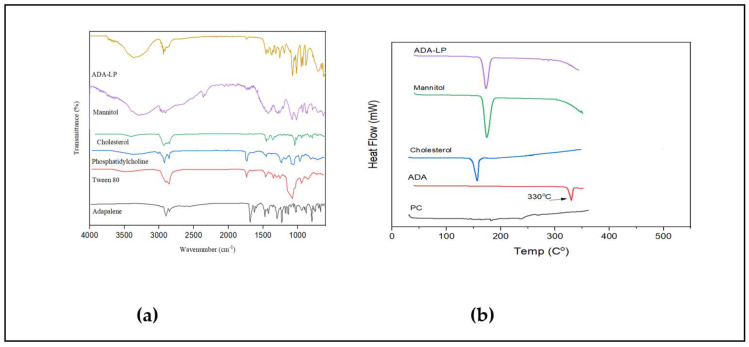
(**a**) FTIR and (**b**) DSC of excipients, drug and prepared ADA-LP.

FTIR spectra shows sharp peaks of C–H bond at 2899 cm^−1^ and of C=O 1686 cm^−1^ in the case of ADA as reported in the literature. To improve the shelf-life of liposomes and to obtain the liposomal formulation in solid form for the purpose of characterization, lyophilization was performed. Mannitol was added as a cryoprotectant for the lyophilization of liposomal formulation. As reported, the process of lyophilization can have deleterious effects on the properties of liposomes including a significant increase in size and drug leakage. Mannitol was added in a concentration of 10% [21]. The peaks of O–H bond, and C–H bond can be seen in the FTIR spectra of mannitol. The characteristic peaks of the drug are absent in the spectra of ADA-LP confirming the encapsulation of drug, as shown in Figure 3a. Similar results were reported in the literature [22]. DSC thermogram shows a sharp peak of ADA at 330 °C, which is the melting point of ADA, confirming the crystalline nature of the drug [15]. The DSC thermogram of ADA-LP, as shown in Figure 3b, has a peak of mannitol between 168 and 170 °C [23]. The complete disappearance of the peak of the drug confirms the change in the nature of ADA, from crystalline to amorphous. It further confirms the entrapment of ADA in liposomes [15]. The entrapment efficiency of ADA-LP was calculated to be 89.69 ± 0.5%, and the loading capacity was 15.32 ± 0.10%. 

### 2.3. Preparation of ADA-LP Gel

To improve the delivery and retention of optimized ADA-LP, drug-loaded liposomal formulation was loaded in gel. 

#### 2.3.1. Characterization of ADA-LP Gel

**Figure 4:**
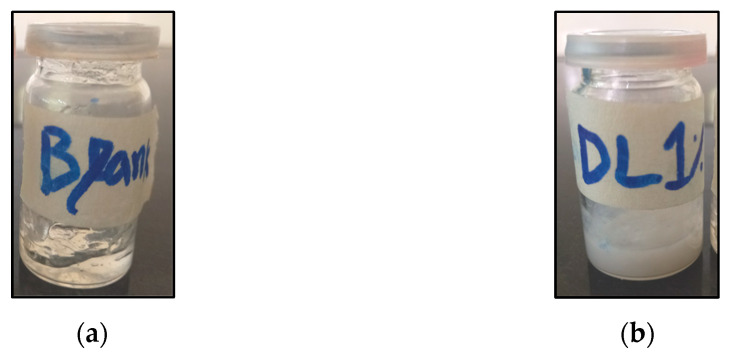
(**a**) 1% Carbopol blank gel. (**b**) Drug-loaded ADA-LP gel in 1% Carbopol.

The prepared blank and ADA-LP-loaded 1% Carbopol 934 were inspected visually. The blank gel was clear, transparent, and smooth in texture, as shown in Figure 4a. ADA-LP gel was also smooth in texture, and no grittiness was noted, as shown in Figure 4b. The spreadability of blank gel was 309 ± 0.08 mm^2^ as compared to ADA-LP gel which was 255.2 ± 0.4 mm^2^. The pH of the final dosage form was 6.43 ± 0.03. The drug content in the ADA-LP gel was 87.083 ± 0.01, as shown in Table 3.

**Figure 5:**
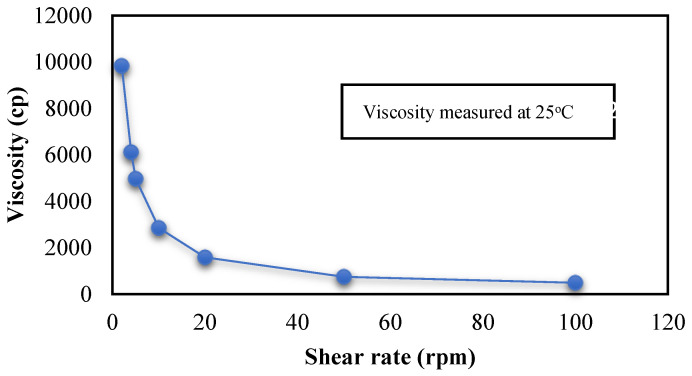
Viscosity of ADA-LP gel measured at 25 °C.

The viscosity of the ADA-LP gel was measured using a Brookfield viscometer. A trend was observed as the increase in the shear rate applied to the gel resulted in a decrease in viscosity. This suggests non-Newtonian or shear thinning behavior as displayed in Figure 5. This helps in easy extrudability and application of gel onto the skin.

#### 2.3.2. In Vitro Release Study

**Figure 6:**
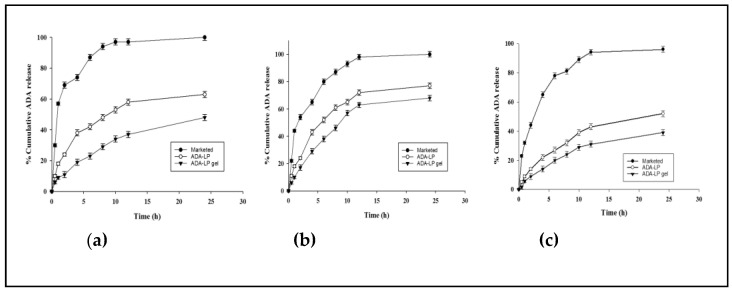
In vitro release study at pH (**a**) 5.5, (**b**) 6.4, and (**c**) 7.4 where *p* = < 0.05.

It was widely reported that the pH of skin with acne lesions is more basic than normal skin. This pH helps in flourishing the *C. acnes* in the skin. Hence, the release pattern was also studied at a pH of 6.4. To facilitate the release at this pH, the pH of the final dosage form, that is, pH of the gel, was also adjusted to 6.4. As the results show, the release was highest at pH 6.4 in comparison to the release at pH 5.5 and 7.4, as shown in Figure 6a,c. The pKa value of ADA is 4.2, which is relatively close to the pH of skin. As the pH increases, the permeability decreases due to the ionization of the drug [24]. The pH of the final dosage form of ADA-LP gel was adjusted to 6.4, which is close to the pH the of skin in AV reported in various studies. Therefore, more drug release was noted at pH 6.4, shown in Figure 6b, as compared to the release at pH 5.5. and 7.4. The marketed formulation released most of its drug content within 10 h regardless of the pH, whereas ADA-LP gel exhibited a controlled release pattern with 72% release within 24 h.

In all cases, there is a gradual increase in the release of ADA until a certain point, after which the release is constant. This can be explained by the formation of nano-emulsion within the system as time increases resulting in a significant decrease in the release of ADA from ADA-LP and ADA-LP gel [25]. Different kinetic models were applied to determine the best fit model for the release study. For both ADA-LP and ADA-LP gel, the Peppas-Sahlin model fit best with an R^2^ value of 0.9963 for ADA-LPs and 0.9900 for ADA-LP gel. This model states that the release is a two-step mechanism involving the swelling of the polymer which leads to the drug release followed by chain relaxation of the polymer [26].

#### 2.3.3. Ex Vivo Permeability Study

**Figure 7:**
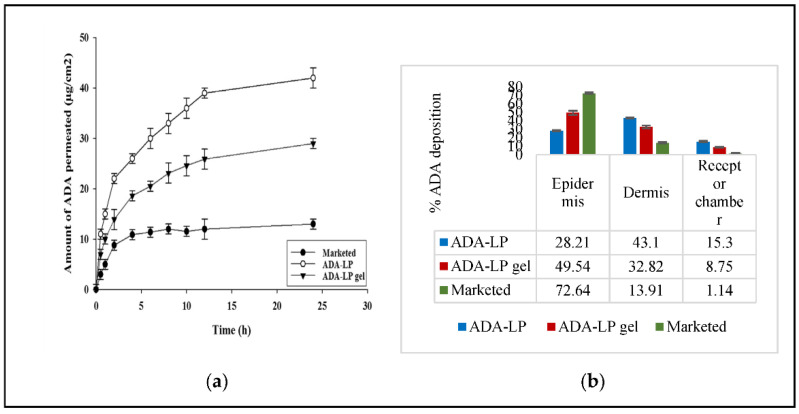
(**a**) Ex vivo permeability of ADA-LP, ADA-LP gel, and marketed gel where *p* = <0.05 and (**b**) Drug deposition study in different skin layers.

Ex vivo permeability was done using Franz Diffusion cell by taking skin from the mice. To mimic real-life conditions, the skin of mice with induced acne was used in the study. The amount of ADA permeated was calculated for drug-loaded liposomes, gel, and marketed gel. The amount of ADA permeated in all cases is shown in Figure 7a. Although ADA is highly lipophilic, it does not permeate the skin, and the amount of ADA permeated is negligible as reported earlier [15]. However, some of the drug was able to permeate the skin since the epidermal barrier is disrupted in AV as the inflammation increases in the region [27]. Moreover, Tween 80, used as surfactant in the current study, has been reported as a penetration enhancer and may have a role in increasing the permeation across skin [28].

The highest amount was permeated for ADA-LP, which was 43 ± 0.06 µg/cm^2^ within 24 h. The skin disposition study was done using adhesive tapes. It was reported that the first 10 strips of adhesive tape separate the epidermis from the skin samples. The data obtained from the study shows that the maximum amount was deposited in the case of ADA-LP gel in the dermis. Furthermore, the marketed gel was found to be only 12% in the dermis and most (72%) remained on the upper layer of skin, as shown in Figure 7b.

#### 2.3.4. Skin Irritation Study

**Figure 8:**
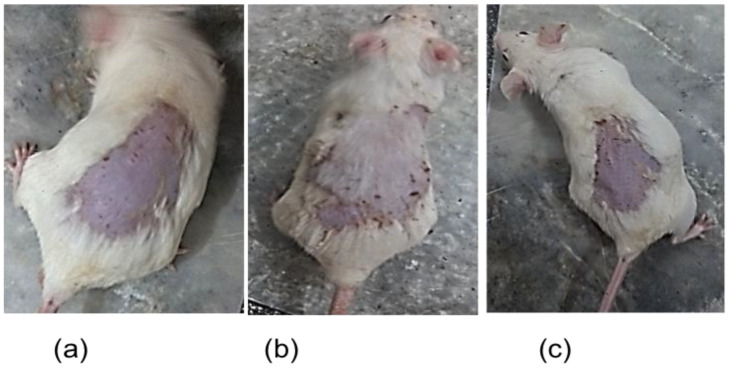
Skin irritation study (**a**) positive control (**b**) negative control and (**c**) treatment with ADA-LP gel.

Any changes in the skin were noted after the application of ADA-LP gel on to the skin of a mouse. The Draize scoring system was used to identify any induced erythema and edema on different time intervals, as shown in Table 4. The results are also displayed in Figure 8, where it can be observed that the negative control has significant erythema as compared to the one treated with ADA-LP gel and positive control. The results indicate that the prepared gel was a non-irritant without any visible changes in the skin and an erythema grading scale of 0.

#### 2.3.5. In Vivo Testosterone-Induced Acne Model

**Figure 9:**
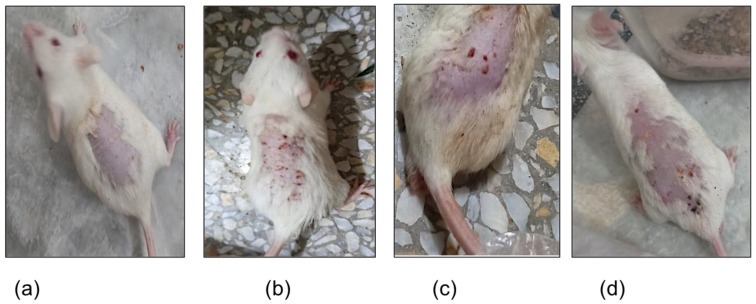
Testosterone-induced acne model in (**a**) normal group, (**b**) negative group, (**c**) positive group, and (**d**) treatment group after 3 weeks.

An ethanolic solution of testosterone (2%) was used to induce acne lesions. After the completion of the third week of study, acne lesions were successfully induced, as shown in Figure 9. The mice in the normal group had no acne lesions or inflammation as expected and can be seen in Figure 9a. The mice in negative, positive, and treatment groups developed acne lesions. A relatively mild form of acne, with visible acne lesions, at the site of application of the testosterone solution can be observed in the negative group in Figure 9b. Papules, in the form of inflamed, red bumps, can be noted in the positive group seen in Figure 9c. Mice in the treatment group had lesions, with inflamed nodules connecting under the skin, as shown in Figure 9d. 

**Figure 10:**
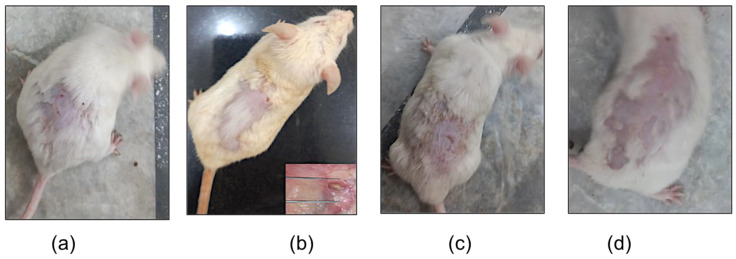
Testosterone-induced acne model in (**a**) normal groups, (**b**) negative group, (**c**) positive group, and (**d**) treatment group at the end of the study.

Since the mice in the normal group had no intervention, no changes in the skin were observed, as shown in Figure 10a. The mice in the negative group only received the testosterone solution throughout the duration of the study, and the progression of the acne towards a more severe form of cystic acne can be observed in Figure 10b. There were visible lesions on the skin of the mice in the positive group; however, a decrease in inflammation was observed, as shown in Figure 10c. A significant change in the lesions was observed in the mice of treatment groups with no visible acne lesions, as shown in Figure 10d.

**Figure 11:**
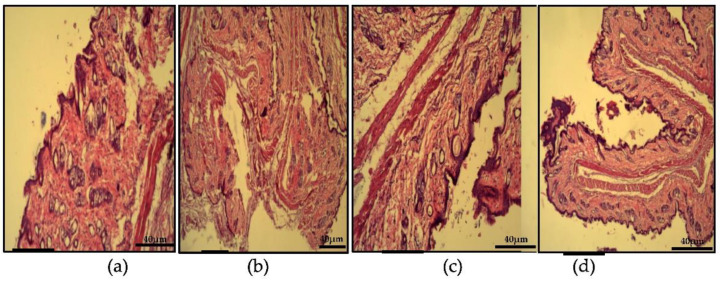
Histopathological analysis of (**a**) normal group, (**b**) negative group, (**c**) positive group, (**d**) treatment group.

An intact epithelium was observed in the normal group, as shown in Figure 11a, The histopathological analysis showed a keratin plug and severe inflammation in the sebaceous glands, as displayed in Figure 11b. The follicle infundibulum is surrounded by purple-stained eosinophils. Abscess with severe inflammation in some of the shafts can also be seen. Signs of inflammation can be observed in the positive control group, as shown in Figure 11c as well as visible acne lesions. The mice in the treatment group had no visible acne lesions, as shown in Figure 10d, but inflammation can be noted. This suggests the progress towards healing of acne lesions and that application of ADA-LP gel for periods longer than 2 weeks could completely heal lesions earlier noted [29]. 

#### 2.3.6. Stability Study

A stability study for the prepared liposomes, ADA-LP, was done according to the International Council for Harmonisation of Technical Requirements for Pharmaceuticals for Human Use (ICH) guidelines over a period of 3 months as presented in Table 5. Liposomes were stable with a slight increase in the size, PDI, and zeta potential. When stored at a temperature of 25 ± 2 °C and a relative humidity of 60% RH ± 5% RH, the size of liposomes increased from 171.5 ± 4.1 to 174.9 ± 3.6 nm, zeta potential from −35.1 ± 1.5 to −37.1 ± 1.1 mV, PDI from 0.123 ± 1.7 to 0.217 ± 1.3. Slight leakage of drug was noted as the entrapment efficiency decreased from 89.69 ± 0.5% to 85.71 ± 0.1%. Moreover, a similar trend was noted with storage conditions of 40 ± 2 °C and a relative humidity of 75% RH ± 5% RH; the particle size of the liposomes increased to 179.8 ± 1.9 nm, zeta potential increased to −39.4 ± 2.3 mV, PDI increased to 0.243 ± 1.5, and EE increased to 83.19 ± 0.2%

## 3. Conclusions

Liposomes (LP) were successfully prepared using a novel SPT method using biocompatible materials such as phosphatidylcholine and cholesterol. Prepared liposomes were then optimized based on their size, PDI, and zeta potential to ensure stability and optimum size. Carbopol-934-based gel was used to load ADA-LP for an efficient delivery of the drug to the skin site. Gel was characterized based on pH, drug content, spreadability, and viscosity. The ADA-LP gel was stable based on physical characteristics, drug content, and pH. In vitro drug release showed the release is maximum at the pH of acne lesions, that is, 6.4 and followed by the Peppas-Sahlin model with sustained release from liposomes. An ex vivo permeability study demonstrated the drug was able to permeate deeper into skin as compared to the marketed formulation. In vivo analysis was done via a testosterone-induced animal model. AV was developed, and the prepared ADA-LP-gel successfully treated the acne with no visible lesions as compared to the marketed formulation. The development and treatment of induced acne lesions was further confirmed by H&E staining. Therefore, it can be concluded that the prepared lipid-based nanocarrier system provides an efficient system for the delivery of the drug and effective treatment of AV.

## 4. Materials and Methods

### 4.1. Materials

Adapalene (gifted from Valor pharmaceuticals, Islamabad, Pakistan, Tween 80, Phosphatidylcholine S100 was kindly gifted by Lipoid, cholesterol, chloroform, Carbopol 934, triethanolamine, ethanol, mannitol, tetrahydrofuran (THF), potassium dihydrogen phosphate, and sodium hydroxide (NaOH) was purchased from Sigma-Aldrich, Taufkirchen, Germany. Testosterone was purchased from Bayer, Karachi, Pakistan.

### 4.2. Preparation of ADA-LP

Liposomes were prepared using a modified spontaneous transition method (SPT) as reported in the literature [5]. Firstly, 6.7495 mg of cholesterol, 7.07 mg of lipid, and different concentrations of ADA (0.3, 0.6, and 1.2 mg) were weighed accurately and dissolved in 3 mL of chloroform. We weighed 12 mg of Tween 80 in a 20 mL vial, and 12 mL of distilled water was added to it. The 0.1% Tween 80 solution was prepared by allowing the vial containing water and weighed Tween 80 to mix on a hotplate stirrer while keeping the speed at 200 rpm. Organic and aqueous phases were added to the flask of a rotary evaporator (Heidolph, Schwabach, Germany). The temperature was set to 45 °C and operated at a speed of 150 rpm for 20 min until the chloroform evaporated. The flask was removed after 20 min, and the obtained nanosuspension was stored in a vial at a temperature of 5 °C. Based on the entrapment efficiency and stability, one nanosuspension was used for further studies. LP were prepared using the same method but without the addition of ADA.

#### 4.2.1. Optimization of LP

A number of factors such as the concentration of PC, surfactant, cholesterol, and varying ratio of organic to aqueous phase can greatly affect the properties of the liposomes such as size and stability. Therefore, different concentrations and ratio of these factors for the synthesis of liposomes were optimized using Box-Behnken design of Design Expert software version 12.0. For the optimization of liposomes in terms of particle size, PDI, and zeta potential, response surface methodology (RSM) was used. The software generated 29 runs employing Box-Behnken. This model offers maximum randomization. Runs with different varying factors and responses are given in Table 2. Each run with specified concentrations of PC, Tween 80 solution, and cholesterol was formulated and evaluated on the basis of size, PDI, and zeta potential.

#### 4.2.2. Characterization of ADA-LP

A zetasizer (Malvern, Nano ZS-90, Worcestershire, UK) was utilized for analyzing the particle size, zeta potential, and PDI of all 29 formulations. Briefly, 10 µL of the sample was diluted with distilled water up to 1 mL in an Eppendorf tube [30]. The compatibility of excipients such as cholesterol, lipid, surfactant, and drug along with ADA-LP were lyophilized, and powdered samples were analyzed based on their bonds via FTIR spectrophotometer (Perkin Elmer, Waltham, MA, USA). For differential scanning calorimetry (DSC), a total of 2 mg of the sample was placed in the aluminum pan, and a DSC thermogram was obtained by heating the sample in range from 30 to 350 °C with an increase in heating at a rate of 10 °C/min [31]. An empty reference pan was used as a control. Samples analyzed using this method included CH, PC, mannitol, ADA, and ADA-LP. Encapsulation efficiency (EE) and loading capacity (LC) were determined by the centrifugation of nanosuspension, and the supernatant was separated and analyzed at 320 nm using a UV-visible spectrophotometer (Dynamica, Halo DB-20, London, UK).

### 4.3. Preparation of ADA-LP Gel

Based on entrapment efficiency, a calculated volume of ADA-loaded nanosuspension (50 mL) was centrifuged at 15,000 rpm for 1 h at −4 °C. The pellets were obtained after removing the supernatant. Pellets were vortexed to form a slurry. For the 1% gel, 30 mg of Carbopol 934 were weighed accurately. It was then soaked in 3 mL of distilled water while stirring at 200–300 rpm. The slurry was added to the mixture. Triethanolamine (TEA) was added to neutralize the solution and form a gel.

The pH of the formulation was adjusted to 6.4. The concentration of ADA was 0.3% *w*/*w* in the prepared gel.

#### 4.3.1. Characterization of ADA-LP Gel

The prepared ADA-LP gel was inspected visually for any grittiness, uniform distribution, color change, or phase separation. The pH of the gels was checked using a pH meter. Glass electrode was immersed directly into the gels, and pH was recorded three times. Average pH was calculated with standard deviation. The viscosity of the gel was measured using a Brookfield viscometer at different shear rates. A total of 10 g of gel was accurately weighed and taken in a beaker. Viscosity was measured with the help of spindle S63 at 2, 4, 5, 10, 20, 50, and 100 rpm. Readings were taken by maintaining a temperature of 25 °C throughout the experiment. Spreadability is the measurement of the ease with which gel can be applied topically. It allows uniform distribution [32]. Spreadability was measured using the glass slide method. Two glass slides were used. The center of one slide was marked with a circle of 1 cm. Gel weighing 0.5 g was placed in the circle. The second slide was placed on the first one. A weight of 500 g was placed on the upper slide, and an increase in the diameter was measured [33]. The following formula was used to calculate the spreadability of gel:

Si = d^2^ × π/4 (1)

where Si is the area in mm^2^ and d is the diameter of spread gel.

For the determination of dug content in the finalized 1% Carbopol 934 gel, 1 g of gel was dissolved in 10 mL of solution of phosphate buffer (pH 6.4) and THF in the ratio of 1:1. The solution was allowed to stir on a hotplate. Mixture was filtered through a 0.45 µm syringe filter and analyzed on UV-visible spectrophotometer at 320 nm [34]. Final drug content was calculated with the following formula:

(2) $$\mathrm{Drug}\;\mathrm{content}\;(\%)=\frac{Actual\;amount\;of\;drug\;detected}{Theoretical\;amount\;of\;drug\;in\;formulation}\;\times \;100\;$$

#### 4.3.2. In Vitro Release Study

In vitro release was performed in a shaking water bath where the temperature was maintained at 37 °C. Three different samples were studied: ADA-LP, ADA-LP gel, and marketed formulation. Each sample had an equal concentration (0.3%) of ADA. The study was done using three different phosphate buffers of pH 5.5, pH 6.4, and pH 7.4. Phosphate buffers and THF were used in the ratio of 1:1 [35]. Dialysis membrane was kept in these buffers for 5–10 min. The membrane bags were tied from one side, all four samples were placed in each bag separately and tied from the other end as well using membrane These membrane bags were then placed in 20 mL vials containing buffer and THF (1:1) as a receiver compartment. The vials were then placed in the water bath. The assembly was then set to shake at 35 strokes/min horizontally. Samples of 1 mL were collected at time intervals of 0, 0.5, 1, 2, 4, 6, 8, 10, 12, and 24 h. To maintain the sink condition, 1 mL of buffer:THF was added. The samples were analyzed on a UV-visible spectrophotometer keeping the buffer:THF solution as a blank. Different kinetic models were applied to study the release pattern of ADA-LP and ADA-LP gel.

#### 4.3.3. Ex Vivo Permeation and Deposition Study

##### Skin Preparation

For the study of ADA permeation, skin from the dorsal region of mice was used. Healthy mice were obtained from the pharmacology lab at Quaid-i-Azam university, Islamabad. A testosterone in vivo model was induced in these mice in order to study the permeation of ADA through disease-induced skin. These mice were sacrificed, and their skin was separated. Hair was removed with help of a razor and the skin was kept at 60 °C in the oven to remove the excess fat. The skin was stored in 1% formalin solution at −25 °C in a freezer. Before conducting the study, the skin was thawed and placed in simple buffer media (pH 6.4) for 30 min.

##### Ex Vivo Permeation Study

The skin with an area of 2 cm^2^ was mounted on the cells of the Franz Diffusion apparatus. The permeability od ADA was evaluated in three mediums with pH of 5.5, 6.4, and 7.4, respectively. The receiving medium was buffer and THF in the ratio of 1:1. The skin was placed in a way so that the stratum corneum faced the donor compartment. Four samples of ADA solution, ADA NS, ADA NG, and marketed formulation of each of concentration 0.3% were injected into the donor compartment. Samples of 1 mL were collected at the interval of 0, 0.5, 1, 2, 4, 6, 8, 10, 12 h. With each withdrawal, 1 mL of a mixture of buffer and THF was added to maintain the sink condition. The samples collected were analyzed at 320 nm on UV visible spectrophotometer. The skin was removed and used for further studies. The ADA permeation per cm^2^ was calculated using following equation [36]:

(3) $$\mathrm{Qn}=\frac{Vr.Cn+\sum_{t=0}^{n-1}Vs.Ct}{A}$$

where, Qn is the amount of drug permeated at the nth time (µg/cm^2^), *Cn* is the concentration of drug in receptor compartment at nth time, Ci is the concentration of the drug in receptor medium at sample ith, *Vr* is the volume media present in receptor compartment, *Vs* is the volume of the sample taken at different time intervals, and *A* is the area. 

##### Drug Deposition Study

The skin was collected after the ex vivo permeability study was cleaned with cotton swabs. The skin was stripped with the help of 10 adhesive tapes of a specific length. The drug contents on the tapes were dissolved in a mixture of buffer and THF. This blend was filtered by passing through a 0.45 µm membrane filter and was analyzed on UV visible spectrophotometer. The rest of the skin was washed with buffer and THF mixture to remove excess drug. It was then chopped, and small pieces were immersed in a solvent blend of buffer and THF for a few hours. Later, it was centrifuged, and the supernatant was separated for further analysis of drug content using UV-visible spectrophotometer at 320 nm.

#### 4.3.4. Skin Irritation Study

Draize patch test [37] was used for studying the possible irritation on the site of application. Swiss albino mice were used in the study. The dorsal side of the mice was shaved with a razor. The mice were kept under observation for any induced injury for 24 h. A total of 0.5 g of ADA-LP gel was applied on the shaved site the next day. Positive control did not receive any treatment, whereas 0.8% formalin solution was applied to negative control [38]. The signs of erythema and edema were observed at different time intervals. The Draize scoring system was used ranging from 0–4, where 0 represents no erythema and edema and 4 being the severe form of erythema and edema.

#### 4.3.5. In Vivo Testosterone-Induced Acne Model

Androgens, including testosterone, are associated with acne [39]. Testosterone was used to induce acne lesion in Swiss albino mice as reported previously in a number of publications [40]. The study was conducted after approval from the Bio-Ethical Committee (BEC) Quaid-i-Azam University Islamabad, Pakistan under reference no. (#BEC-FBS-QAU2022–423). The mice were divided into groups of four consisting of the normal group, negative control, positive control, and the treatment group. Positive, negative, and treatment groups received only testosterone solution until the induction of acne lesions. Moreover. The normal group did not receive any kind of treatment or intervention and were only fed for the whole study. The normal group was also kept separate from other groups. Hair was removed from the dorsal side of mice. They were kept under observation for 24 h for any development of erythema or irritation. The 2% testosterone solution in ethanol was applied on the shaved side every morning and evening for 3 weeks. After induction of acne, the negative group received testosterone solution, positive group was applied marketed gel (0.3%), and the treatment group was applied liposomal gel in the morning and testosterone solution in the evening [41].

#### 4.3.6. Stability Study

ICH guidelines {Q1A(R^2^)} were followed while performing the stability study [42]. The stability of prepared ADA-LP formulation was performed on two storage conditions, over a period of 6 months. Accelerated storage conditions for general substances with a temperature of 40 °C ± 2 °C, relative humidity of 75% RH ± 5% RH, and accelerated storage conditions for drugs intended to be stored in refrigerator with a temperature of 25 °C ± 2 °C and at 60% RH ± 5% RH were used. The change in the particle size, zeta potential, and PDI were noted over the period of 6 months at different time intervals (0, 1, 3, 6 months).

## Figures and Tables

**Table 1:** Design summary for the optimization of LP.

Design Summary					
File Version	12.0.3.0	Subtype		Randomized	
Study Type	Response Surface	Runs		29	
Design Model	Quadratic	Blocks		No Blocks	
Build Time (ms)	19.00				
Factor	Name	Units	Type	Minimum	Maximum
A	Phosphatidylcholine (PC)	mg	Numeric	1.40	7.07
B	Cholesterol (CH)	mg	Numeric	1.90	11.60
C	Tween 80 solution	%	Numeric	0.1	1.0
D	Organic/aqueous ratio		Numeric	2.00	6.00

**Table 2:** Optimization of LP using Design Expert.

	Independent Variable				Dependent Variables		
Run	Factor 1: PC (mg)	Factor 2: CH (mg)	Factor 3: Tween 80 Solution (%)	Factor 4: Organic/Aqueous Ratio	Response 1: Size (nm)	Response 2: Zeta Potential (mV)	Response 3: PDI
1	7.07	1.90	0.55	1:4	267.7 ± 2.4	−19.0 ± 1.4	0.48 ± 0.7
2	7.07	11.59	0.55	1:4	313.2 ± 3.6	−34.6 ± 1.6	0.64 ± 1.4
3	7.07	6.74	0.55	1:2	280.7 ± 1.2	−15.2 ± 2.1	0.37 ± 1.1
4	4.23	11.59	0.10	1:4	396.8 ± 4.3	−27.0 ± 1.1	0.60 ± 1.6
5	4.23	11.59	0.55	1:6	273.9 ± 3.2	−26.3 ± 1.9	0.49 ± 1.3
6	7.07	6.74	0.55	1:6	282.4 ± 4.7	−19.9 ± 1.4	0.41 ± 2.8
7	4.23	1.90	0.55	1:2	296.7 ± 1.7	−12.1 ± 2.7	0.37 ± 2.5
8	4.23	6.74	0.10	1:6	241.5 ± 2.5	−20.6 ± 1.5	0.47 ± 1.8
9	1.40	1.90	0.55	1:4	251.8 ± 2.8	−7.32 ± 1.3	0.46 ± 1.2
10	4.23	6.74	0.55	1:4	280.2 ± 3.2	−27.0 ± 1.3	0.45 ± 1.1
11	4.23	6.74	0.55	1:4	277.4 ± 4.5	−27.0 ± 1.5	0.41 ± 1.9
12	4.23	6.74	1	1:2	318.3 ± 1.9	−21.6 ± 1.6	0.58 ± 2.7
13	1.40	6.74	1	1:4	485.5 ± 2.5	−18.5 ± 2.3	0.68 ± 2.5
14	1.40	6.74	0.55	1:2	458.6 ± 4.6	−24.2 ± 2.4	0.67 ± 2.9
15	1.40	11.59	0.55	1:4	493.4 ± 2.4	−26.6 ± 1.9	0.67 ± 1.2
16	4.23	6.74	0.55	1:4	277.4 ± 2.2	−27.0 ± 2.4	0.41 ± 1.6
17	4.23	11.59	0.55	1:2	327.2 ± 1.3	−26.4 ± 2.6	0.64 ± 1.3
18	4.23	6.74	0.55	1:4	202.7 ± 5.4	−21.4 ± 1.5	0.31 ± 3.9
19	4.23	6.74	0.55	1:4	184.6 ± 2.3	−27.0 ± 1.7	0.45 ± 3.2
20	4.23	6.74	1	1:6	355.4 ± 4.4	−25.1 ± 1.3	0.53 ± 1.4
21	1.40	6.74	0.1	1:4	434.6 ± 3.3	−17.4 ± 2.6	0.53 ± 1.8
**22**	**7.07**	**6.74**	**0.1**	**1:4**	**156.1 ± 2.6**	**−26.3 ± 1.5**	**0.12 ± 1.3**
23	4.23	1.90	0.1	1:4	298.7 ± 4.6	−11.3 ± 2.4	0.53 ± 2.1
24	1.40	6.74	0.55	1:6	432.2 ± 2.9	−20.4 ± 1.5	0.64 ± 3.8
25	4.23	11.59	1	1:4	315.8 ± 3.5	−24.2 ± 1.7	0.52 ± 2.4
26	7.07	6.74	1	1:4	237.5 ± 3.3	−19.0 ± 1.1	0.40 ± 2.0
27	4.23	1.90	0.55	1:2	296.7 ± 2.8	−12.1 ± 2.3	0.37 ± 1.5
28	4.23	6.74	0.1	1:6	320.0 ± 2.1	−23.0 ± 3.5	0.45 ± 1.2
29	4.23	1.90	1	1:4	240.0 ± 2.5	−22.0 ± 1,8	0.35 ± 1.8

**Table 3:** Characterization of Blank and ADA-LP Gel.

Characterization of Blank and ADA-LP Gel (Mean ± SD, Where n = 3)				
Dosage Form	Physical Appearance	pH	Spreadability (mm^2^)	Drug Content (%)
Blank gel	Clear	6.4 ± 0.06	309 ± 0.08	-
ADA-LP gel	Opaque	6.43 ± 0.03	255.2 ± 0.4	87.083 ± 0.01

**Table 4:** Skin irritation study according to Draize scoring system.

	Time (h)	Positive Control	Negative Control	Treatment with ADA-LP Gel
Erythema	1	0	2	0
	12	0	4	0
	24	0	4	0
Edema	1	0	1	0
	12	0	1	0
	24	0	1	0

**Table 5:** Stability study done over a period of 6 months.

	25 ± 2 °C at 60% RH ± 5% RH					40 ± 2 °C at 75% RH ± 5% RH				
**Time (Months)**	0	1	2	3	6	0	1	2	3	6
**Size (nm)**	171.5 ± 4.1	171.9 ± 2.1	173.4 ± 1.7	174.7 ± 1.6	174.9 ± 3.6	171.5 ± 4.1	173.9 ± 3.2	175.1 ± 2.6	176.4 ± 1.5	179.8 ± 1.9
**Zeta** **potential (mV)**	−35.1 ± 1.5	−35.6 ± 1.2	−36.3 ± 2.7	−36.7 ± 2.1	−37.1 ± 1.1	−35.1 ± 2.1	−36.9 ± 2.7	−37.9 ± 3.6	−38.1 ± 2.7	−39.4 ± 2.3
**PDI**	0.123 ± 1.7	0.127 ± 2.2	0.212 ± 1.5	0.214 ± 2.3	0.217 ± 1.3	0.123 ± 1.7	0.195 ± 1.5	0.236 ± 1.3	0.233 ± 2.7	0.243 ± 1.5
**EE%**	89.69 ± 0.5	88.20 ± 0.4	86.12 ± 0.3	86.91 ± 0.1	85.71 ± 0.1	89.69 ± 0.5	87.31 ± 0.7	84.06 ± 0.1	84.63 ± 0.9	83.19 ± 0.2

## Data Availability

The data are included in the manuscript.
